# Home-Based Cognitive Intervention for Healthy Older Adults Through Asking Robots Questions: Randomized Controlled Trial

**DOI:** 10.2196/47229

**Published:** 2024-04-22

**Authors:** Seiki Tokunaga, Takuya Sekiguchi, Kumi Watanabe Miura, Hikaru Sugimoto, Masato S Abe, Kazuhiro Tamura, Taishiro Kishimoto, Takashi Kudo, Mihoko Otake-Matsuura

**Affiliations:** 1 Center for Advanced Intelligence Project, RIKEN, Tokyo, Japan; 2 Faculty of Culture and Information Science, Doshisha University, Kyoto, Japan; 3 Department of Neuropsychiatry, School of Medicine, Keio University, Tokyo, Japan; 4 Department of Psychiatry, Graduate School of Medicine, Osaka University, Osaka, Japan

**Keywords:** cognitive intervention, home-based experiment, robots, older adults, technology adoption, digital health

## Abstract

**Background:**

Asking questions is common in conversations, and while asking questions, we need to listen carefully to what others say and consider the perspective our questions adopt. However, difficulties persist in verifying the effect of asking questions on older adults’ cognitive function due to the lack of a standardized system for conducting experiments at participants’ homes.

**Objective:**

This study examined the intervention effect of cognitive training moderated by robots on healthy older adults. A focus on the feasibility of the intervention at participants’ homes was also maintained. Feasibility was evaluated by considering both the dropout rate during the intervention and the number of questions posed to each participant during the experiment.

**Methods:**

We conducted a randomized controlled trial with 81 adults older than 65 years. Participants were recruited through postal invitations and then randomized into 2 groups. The intervention group (n=40) received sessions where participants listened to photo-integrated stories and posed questions to the robots. The control group (n=41) received sessions where participants listened to photo-integrated stories and only thanked the robots for confirming participation. The participants participated in 12 dialogue sessions for 2-3 weeks. Scores of global cognitive functioning tests, recall tests, and verbal fluency tasks measured before and after the intervention were compared between the 2 groups.

**Results:**

There was no significant intervention effect on the Telephone Interview for Cognitive Status-Japanese scores, recall tests, and verbal fluency tasks. Additionally, our study successfully concluded with no participant dropouts at follow-up, confirming the feasibility of our approach.

**Conclusions:**

There was no statistically significant evidence indicating intervention benefits for cognitive functioning. Although the feasibility of home-based interventions was demonstrated, we identified areas for improvement in the future, such as setting up more efficient session themes. Further research is required to identify the effectiveness of an improved cognitive intervention involving the act of asking questions.

## Introduction

The aging of the world’s population has led to a growing interest in maintaining a healthy lifestyle and enhancing the quality of life in later years. Previous studies have suggested that a healthy lifestyle can prevent or delay age-related cognitive decline [1,2].

Social interaction is a key component of a healthy lifestyle in later years. The social isolation attributed to social distancing during the COVID-19 pandemic may have particularly impacted older adults. For example, the impact of loneliness due to isolation on mental health is concerning [3].

Another concern is that reduced social participation can increase the risk of cognitive decline [4]. Conversations with a variety of people can be a trigger for understanding others’ perspectives and acquiring new information, which plays an important role in maintaining and improving older adults’ cognitive function [5]. Sharifian et al [6] showed that older adults with a higher proportion of family members in their social networks have less contact with friends, which is negatively associated with their episodic memory. As the aforementioned study conjectured, contact with family members is usually restricted to obligatory tasks. In contrast, contact with friends is more likely to involve new conversations and information exchanges, which may be cognitively beneficial. Thus, cognitive maintenance and improvement mechanisms may be absent for older adults whose social participation is limited, such as those who only communicate with family members or caregivers or those who are completely isolated. The lives of older adults often involve factors that prevent social participation, such as the COVID-19 pandemic [7]. Therefore, methods to safely and remotely deliver cognitive training programs have been developed [8,9]. Dodge et al [8] have reported the effect of improving language-based executive function for older adults who have mild cognitive impairments through discussion intervention. However, previous studies still indicate a need for conversational human partners; hence, full automation of conversations needs further study.

Another issue in providing cognitive training for older adults is the limited available human resources for performance [10]. For example, a software agent that learns user characteristics, such as an intelligent assistant [11], could help older adults manage their health based on personal data collected automatically [12]. Furthermore, socially assistive robots may reduce the burden on caregivers to continuously monitor older adults who live alone with cognitive impairments and are at daily risk of various accidents [13].

Hence, our goal is to develop assistive robots that enable older adults’ remote participation in conversational cognitive training with the same degree of effectiveness as in-person social interaction for cognitive function. Home-based cognitively assistive robots aim to conduct cognitive training for older adults at home. A previous study has suggested that cognitively assistive robots have the potential to benefit older adults and society [14,15]; however, few studies have rigorously evaluated their benefits [16]. The challenge to overcome in promoting such research is the difficulty in controlling users’ characteristics related to speech. For example, depending on personality and familiarity with device use, the amount of conversation with the robot may differ from person to person. Therefore, the training effects provided by the robot cannot be accurately evaluated without controlling for such factors.

Similarly, we developed a conversational intervention program, Photo-Integrated Conversation Moderated by Robots (PICMOR), and examined its effect on healthy older adults’ cognitive function [17]. Briefly, the PICMOR program is a group conversation that uses photos taken by the participants beforehand. The program consists of 2 parts. First, the participants elaborate on the photos. Second, the participants receive questions about the photos from other participants and answer them. Each part has a time limit and is controlled by a robot facilitator. Notably, the questioning time for each participant is controlled by the robot. It has the function of encouraging participants who talk too much or too little to reduce or promote their speech as needed.

In a randomized controlled trial (RCT), we observed the beneficial effects of PICMOR on performance in a letter fluency task [17]. A follow-up experiment using multimodal magnetic resonance imaging provided candidate brain metrics that could be associated with the intervention effects on phonemic verbal fluency [18-20]. For instance, resting-state functional connectivity between the left inferior frontal gyrus, one of the most important brain regions for verbal fluency, and the right temporal pole, a semantic-related brain region, positively correlated with enhanced verbal fluency performance [18]. Moreover, we conducted another RCT using PICMOR and examined whether the intervention effects on verbal fluency varied as a function of neuronal states estimated from blood-based biomarkers, such as plasma neurofilament light chain [21]. The results showed that individuals with lower neurofilament light chain, indicating a relatively intact neuronal state, performed better in a category fluency task.

Despite these observed benefits, we could not accurately identify the components of this intervention program that contribute to the enhancement of verbal fluency [18]. This is because the intervention methodology included a variety of cognitive and mental activities, such as preparing a short presentation within a certain length of time, flexibly asking and answering questions among participants, intentionally storing and manipulating information to ask questions, and refraining from interrupting other participants’ utterances.

As merely developing methods to improve performance on specific tasks is not enough to improve cognitive function generally applicable to daily life, intervention strategies that bundle multiple components are being researched [22-25]. However, distinguishing these components to clarify the mechanisms underlying the intervention effects and develop more effective intervention methods is also important. In this study, we explored the effects of “asking questions” on cognitive functions among healthy older adults, assuming that it would be an important factor in verbal fluency enhancement.

This study conducted an RCT to collect evidence on the feasibility of asking questions to robots at home and its effect on the cognitive functions of healthy older adults. Our hypothesis is that cognitive function will improve in the intervention group compared to the control group. Additionally, the effects and future improvements of the intervention program are discussed.

## Methods

### Trial Design

**Figure 1.**
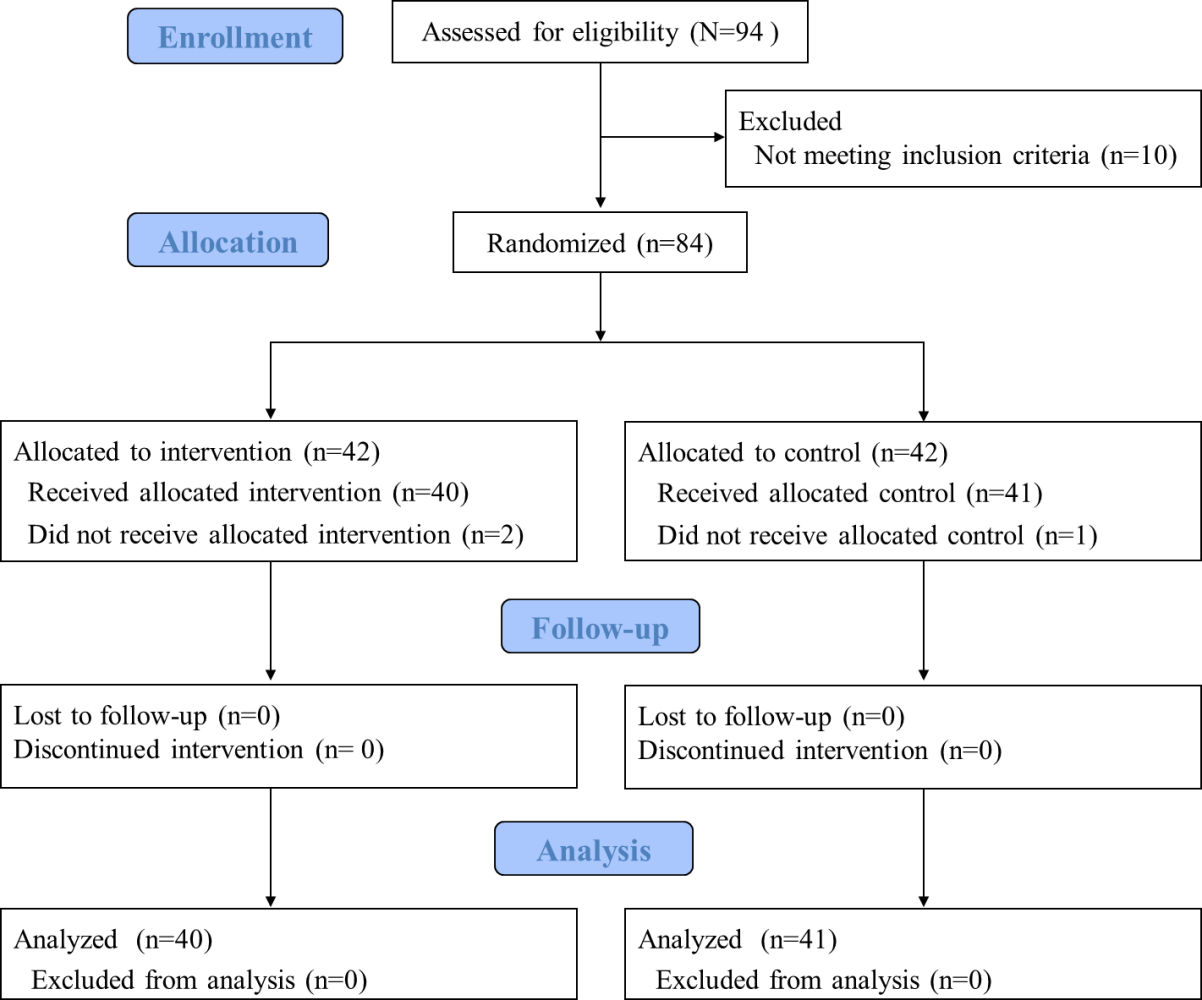
The CONSORT (Consolidated Standards of Reporting Trials) diagram flowchart.

This study used an RCT with a two-parallel-arm design and 1:1 allocation. All RCT procedures were conducted from February to November 2021 at the participants’ homes. Figure 1 presents the CONSORT (Consolidated Standards of Reporting Trials) flowchart of this trial. No participants dropped out of the intervention at follow-up.

### Ethical Considerations

The studies involving human participants were reviewed and approved by the RIKEN Ethical Committee. All participants provided written informed consent to participate in the study. The study is registered with ClinicalTrials.gov (UMIN000039489).

### Participants

Participants were community-dwelling older adults with subjective memory concerns living in an urban city (Wako-shi) in Japan. They were recruited through postal invitations. In total, 92 participants were screened for eligibility. The eligibility criteria for the trial were as follows: (1) age ≥65 years, (2) Telephone Interview for Cognitive Status-Japanese (TICS-J) score ≥33, and (3) complaints of cognitive concern. The exclusion criteria were as follows: (1) any neurological impairment known to affect the central nervous system, (2) any serious complicating disorder, (3) any history of serious head injury, (4) any disease or medication known to affect the central nervous system, (5) medical history of stroke, and (6) need for care. We defined the term “need for care” as certification of care needs levels or “support need levels” in the Japanese public long-term care insurance system, and participants were screened based on their self-report.

### Intervention Design

In the intervention group, participants listened to stories and subsequently asked the dialogue robots as many questions as possible during the experiment. In the control group, participants listened to the stories and subsequently offered short greetings to the robots as evidence of participation. Participants received 2-3 intervention sessions weekly; we arranged the schedules with participants individually to complete 12 dialogue sessions. The theme of each session was based on our previous studies [17,26]. The training and intervention procedure is described as follows: before the experiment, all the experimental devices were mailed to each participant. The system was preliminarily set up for each participant, eliminating the need for them to log in to the system. On the first day of the experiment, participants learned how to use the robot and tablets through Zoom. Then, a practice session was conducted via Zoom. In addition, 2 sessions were conducted with the administrator’s face hidden so that participants could become familiar with the devices. Finally, the participants were asked to answer questionnaires on the last day. For other experimental dates, participants received an intervention program.

**Figure 2.**
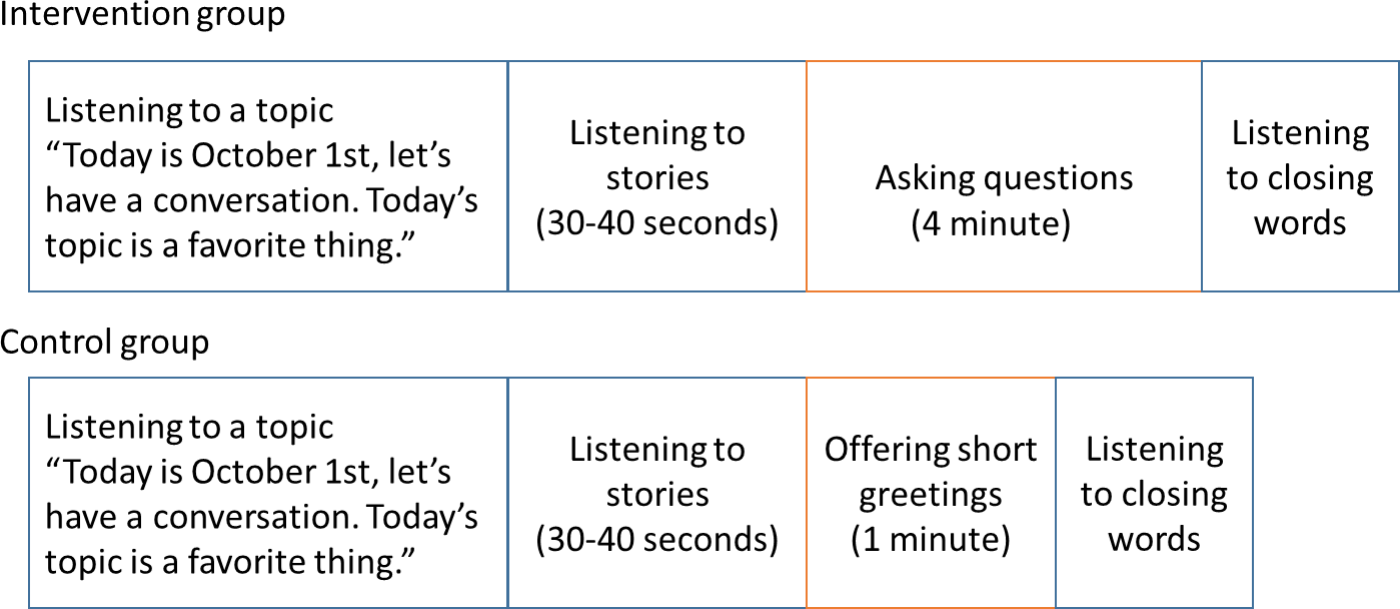
Session timelines for the intervention and control groups.

The experimental procedure for both groups included listening to a story and subsequently asking questions to robots while looking at a photo. Both the photos and a summary of the older adults’ conversation (called a story) were preliminarily collected from 2 older adults [27]. For storytelling, the length of the story was adjusted to 30-40 seconds, referring to the logical memory task of the Wechsler Memory Scale-Revised (WMS-R) [28]. In the intervention group, participants posed as many questions as possible within 4 minutes. When the participants asked questions, the robots provided a plausible response from a list of approximately 550 responses collected beforehand. Contrastingly, in the control group, participants indicated their participation by simply saying “Thank you for the conversation” within 1 minute. Participants were required to push a switch before each utterance, as the pushing switch had a trigger function for the robots to activate question-answering mode. Further details are presented in Figure 2. Therefore, the difference between the intervention and control groups was whether questions were asked during the dialogue session.

**Figure 3.**
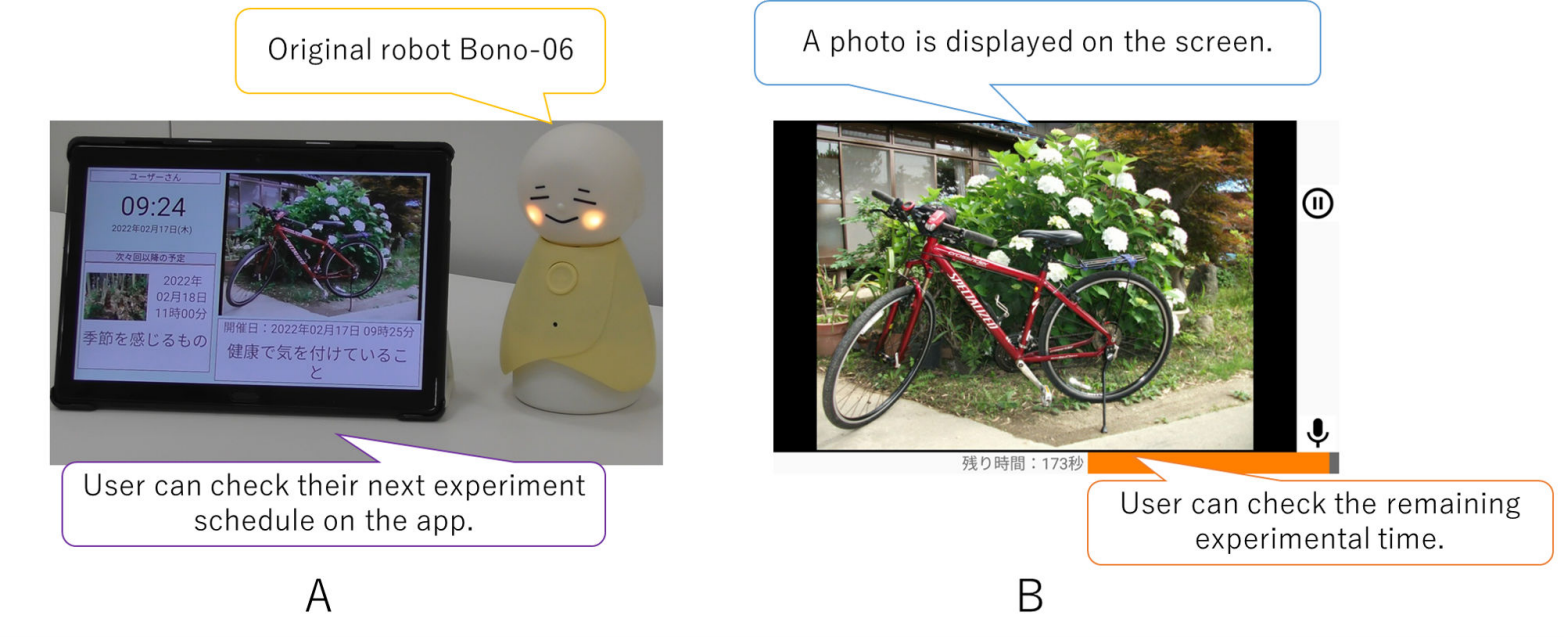
Experimental devices for participants. (A) Android app and original dialogue with robot Bono-06. (B) App screen during the experiment.

For the experimental devices in our experimental setting, we used an original robot called Bono-06 [29] as a user interface for older adults (shown in Figure 3A). Bono-06 has 1 degree of freedom for nodding its head. Red, green, and blue full-color LEDs on its cheeks indicate the system’s status, such as whether it has successfully connected to a tablet. Additionally, a push switch on the chest allows intuitive interaction with older adults. In this study, the robots were designed to enable older adults to ask them questions by only pushing a switch. We also developed an original app that manages participants’ experimental schedules and displays photos and experimental time in dialogue sessions (shown in Figure 3B). The app was designed to display participants’ experimental schedules and run them automatically so that participants could participate in the experiment by turning on their tablets and robots at home without cumbersome operations, such as taps and swipes.

Finally, the system’s operation in home-based experiments was also considered [26]. A delivery and reporting function was implemented in our system so that the experiment administrator could remotely set up each experimental session as scheduled and observe the system report of each session, including the number of utterances and errors. In addition, the experiment report, including the transcribed audio, namely utterances for each dialogue session, was stored in a database on the cloud server. Thus, the experiment administrator could remotely monitor and download the results. In other words, the administrator monitored the experiment while the actual experiment was being conducted in the participants’ homes.

### Outcome

Our primary concern was to examine the extent to which our intervention program improved cognitive function. The TICS-J [30], recall tests, and verbal fluency tasks [31] were assessed before and after the intervention as primary outcomes by well-trained psychologists.

The TICS-J, an 11-item cognitive test, was used to assess global cognition [30]. TICS-J included the immediate recall test, in which the participants were asked to recall a 10-word list. In addition to that, we also conducted a delayed recall test 5 minutes after the immediate recall test. The numbers of words recalled were the scores of these tests. For comparison with a previous RCT [17], the results of the immediate recall test were also reported separately from the total TICS-J score.

Verbal fluency tests were conducted to assess verbal and executive control abilities. Two types of verbal fluency tasks were performed: letter and category [31]. In the letter fluency task, participants were required to produce as many words as possible, beginning with a given letter (“ka” in Japanese) within 1 minute. In the category fluency task, they were asked to produce as many words as possible belonging to a specific category (animals) within 1 minute. The number of words generated was the score for each task. All tests were conducted via telephone interviews.

As a secondary aim, we also investigated the intervention effect on the suboutcomes using questionnaires, including the World Health Organization’s 26-item Quality of Life questionnaire [32], the Japanese version of the Geriatric Depression Scale (short form) [33], and the Tokyo Metropolitan Institute of Gerontology-Index of Competence [34], to assess quality of life, depression symptoms, and functional capacity.

We also examined the factors of our intervention program that should be improved in the future to increase its effectiveness, which will be mainly reported in the *Ancillary Analysis* section. First, we counted the total number of utterances to measure the extent to which our intervention prompted participants to speak. In this study, the number of turns taken by a participant in a conversation with the robot was defined as the number of utterances. Second, we related it to participants’ cognitive test scores to examine how much the intervention worked differently, depending on their cognitive functions. Third, we investigated the extent to which participants’s use of digital devices affected their total amount of utterances. This was achieved by asking participants to answer a 4-item questionnaire about the frequency of their use of PCs, emails, smartphones, and flip phones in their daily lives (1: usually; 2: sometimes; 3: rare; 4: never).

The reason for the distinction between smartphones and flip phones lies in the history of the cell phone market in Japan [35]. Although flip phones used to be popular in Japan, smartphone use began to exceed that of flip phones in 2013. The difference in the time of popularization between the two could result in different user demographics; in other words, those who still frequently use flip phones may be less likely to switch to a new device compared to those who use smartphones. This has particular implications among older adults, the target population of our study. In fact, in 2020, the percentage of Japanese people in their sixties using flip phones was about 26%, while that of Japanese people in their twenties was about 12%. Considering the possibility that these differences between both users might affect their attitude toward the device used in our intervention, we decided to ask them separately about their use.

### Randomization Implementation

Stratified block randomization with a 1:1 allocation was implemented. Participants were stratified into male and female groups and then sorted based on total TICS-J scores. Subsequently, blocks of size 2 were created and randomized. The coding was performed in R 4.3.2 (R Foundation for Statistical Computing). This determined which participants belonged to the intervention or control group. The experimenter assigned participants to the 2 groups based on this result. The person who conducted the randomization was different from the experimenter and had no information other than IDs, TICS-J scores, and gender at the time of randomization. The assessors were blinded to the allocation results.

### Statistical Analysis

As explained in the *Outcome and Estimation* section, we used linear mixed models with random intercepts to examine the effects of the intervention on cognitive function. The models included total TICS-J scores, immediate and delayed recall test scores, letter fluency test scores, and category fluency scores as outcome variables, with time (1: end point; 0: baseline), group (1: intervention group; 0: control group), and their interaction terms as independent variables. We interpreted the regression coefficients associated with the interaction terms as the degree of the intervention effects. We also reported the sizes of intervention effects measured by *f*^2^ [36].

For the ancillary analysis, we applied linear mixed models with random intercepts to the intervention group, which included cognitive function scores as outcome variables; time, the number of utterances, and their interaction terms as explanatory variables; and gender, age, and education as control variables. For these models, we reported regression coefficients associated with the number of utterances to understand the relationship between cognitive function scores at baseline and the number of utterances; we also reported regression coefficients associated with the interaction term to understand the relationship between the number of utterances and change in scores before and after the intervention. In addition, we reported the relationship between participants’ digital device use in the intervention group and their total number of utterances using 2-tailed *t* tests.

All analyses were performed using R. To implement the linear mixed models, the lmer function in the R package (lme4) was used [37].

## Results

### Baseline Data

A total of 40 participants in the intervention group and 41 in the control group underwent cognitive testing, both at baseline and end point, and were included in the analysis. Table 1 shows the baseline characteristics of the participants. For all demographic and cognitive variables (Table 1 and Table 2), there were no major differences between the intervention and control groups, namely, participant attributes were balanced at baseline.

**Table 1.** Baseline characteristics of the intervention and control groups (N=81).

Characteristics		Intervention (n=40)	Control (n=41)
Age (year), mean (SD)		73.9 (3.8)	74.0 (4.1)
Gender (female), n (%)		24 (60)	25 (61)
Education (≥13 years), n (%)		19 (48)	30 (73)
WHO^a^ QOL26^b^ questionnaire, mean (SD)		3.68 (0.38)	3.67 (0.41)
GDS-15^c^, mean (SD)		2.17 (2.00)	2.05 (2.32)
**TMIG-IC** ^**d**^ **, mean (SD)**			
	Total score	11.93 (1.1)	11.73 (1.3)
	Instrumental activity of daily living	5.00 (0.0)	4.95 (0.2)
	Intellectual activity	3.62 (0.7)	3.73 (0.6)
	Social role	3.30 (0.9)	3.05 (1.0)

a WHO: World Health Organization.

b QOL26: Quality of Life 26-item. For the WHO QOL26, 2 participants who selected multiple items were excluded from the intervention group.

c GDS-15: Geriatric Depression Scale-15.

d TMIG-IC: Tokyo Metropolitan Institute of Gerontology Index of Competence.

**Table 2.** Comparison of cognitive test scores at baseline and end point between the intervention and control groups.

Cognitive test	Intervention (n=40)		Control (n=41)	
	Baseline, mean (SD)	End point, mean (SD)	Baseline, mean (SD)	End point, mean (SD)
TICS-J^a^	36.30 (2.03)	36.80 (2.57)	36.07 (1.82)	37.07 (2.24)
Category fluency	15.47 (4.49)	17.27 (5.09)	16.34 (3.66)	16.78 (4.34)
Letter fluency	13.93 (3.55)	14.43 (3.97)	14.27 (4.14)	14.37 (3.79)
Immediate recall	7.20 (1.40)	7.90 (1.39)	6.80 (1.42)	7.61 (1.46)
Delayed recall	6.30 (1.94)	7.17 (1.82)	5.98 (1.51)	6.95 (1.61)

a TICS-J: Telephone Interview for Cognitive Status-Japanese.

### Outcome and Estimation

Table 3 shows the results of the linear mixed models used to examine the intervention effects on cognitive function. No significant intervention effects were found for the total TICS-J score, immediate and delayed recall, and verbal fluency. The regression coefficient associated with the time-group interaction term for the category fluency test score was the largest among the study outcomes (1.361), but the effect was not significant (*P*=.09; effect sizes of *f*^2^<0.01 for all outcomes). We found no significant intervention effects on any of the secondary outcomes. Table 4 shows the conversation theme of each session and the descriptive statistics for the number of participants’ utterances therein.

**Table 3.** Results of the linear mixed models used to examine the intervention effects on cognitive function.

Category	Time				Group				Time × group			
	Coefficients (95%CI)	SE	*t* (df)^a^	*P* value	Coefficients (95%CI)	SE	*t* (df)^a^	*P* value	Coefficients (95%CI)	SE	*t* (df)^a^	*P* value
TICS-J^b^	1.00 (0.32 to 1.68)	0.35	2.89 (79)	.005	0.23 (–0.72 to 1.17)	0.49	0.47 (127.98)	.64	–0.50 (–1.46 to 0.46)	0.49	–1.02 (79)	.31
Category fluency	0.44 (–0.65 to 1.53)	0.56	0.79 (79)	.43	-0.87 (–2.78 to 1.05)	0.98	–0.88 (108.5)	.38	1.36 (–0.19 to 2.91)	0.79	1.72 (79)	.09
Letter fluency	0.10 (–1.00 to 1.19)	0.56	0.17 (79)	.86	-0.34 (–2.02 to 1.33)	0.86	–0.40 (119.1)	.69	0.40 (–1.16 to 1.96)	0.80	0.51 (79)	.62
Immediate recall	0.80 (0.34 to 1.27)	0.24	3.39 (79)	.001	0.40 (–0.22 to 1.01)	0.32	1.25 (133.8)	.21	–0.10 (–0.77 to 0.56)	0.34	–0.31 (79)	.76
Delayed recall	0.98 (0.41 to 1.54)	0.29	3.38 (79)	.001	0.32 (–0.42 to 1.07)	0.38	0.85 (133.5)	.40	–0.10 (–0.90 to 0.70)	0.41	–0.25 (79)	.81

a Satterthwaite degree of freedom.

b TICS-J: Telephone Interview for Cognitive Status-Japanese.

**Table 4.** Descriptive statistics of the number of utterances per session. The participant count in the intervention group is 40. Mean, SD, minimum, and maximum are at the participant level.

Theme	Total, n	Mean (SD)	Minimum	Maximum
1. Favorite things	291	7.28 (2.20)	1	10
2. Neighborhood landmarks	344	8.60 (1.85)	2	11
3. I try to get off the train at a station that I seldom use	346	8.65 (2.05)	2	11
4. Favorite foods	346	8.65 (1.70)	4	12
5. For my health	335	8.38 (1.23)	5	10
6. Found on a 10-minute walk	363	9.07 (1.94)	4	11
7. Saving energy	317	7.92 (1.67)	4	11
8. Funny stories and mistakes	328	8.20 (1.92)	4	11
9. Things to get rid of	323	8.07 (1.83)	2	11
10. Tips for daily living	353	8.82 (1.80)	3	12
11. Feeling the season	359	8.97 (1.75)	5	12
12. Starting something new	366	9.15 (1.72)	5	12

### Ancillary Analysis

The average total number of utterances of the participants in the intervention group was 101.78 (SD 14.72). The number of utterances was positively correlated with a higher letter fluency score at baseline (letter fluency: B=0.11, SE 0.04; *P*=.01), while no significant associations were found for the other outcomes (TICS-J: B=0.03, SE 0.03; *P*=.30; category fluency: B=0.10, SE 0.06; *P*=.07; immediate recall: B=−0.01, SE 0.02; *P*=.64; delayed recall: B=0.01, SE 0.02; *P*=.55).

There was no significant association between the number of utterances and change in scores for any of the outcomes (TICS-J: B=−0.03, SE 0.03; *P*=.31; category fluency: B=−0.01, SE 0.04; *P*=.79; letter fluency: B=0.02, SE 0.03; *P*=.63; immediate recall: B=−0.003, SE 0.02; *P*=.85; delayed recall: B=−0.01, SE 0.02; *P*=.76).

**Figure 4.**
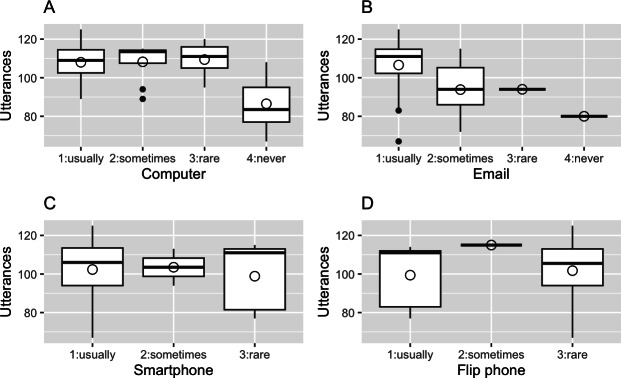
Box plots for the relationship between the frequency of device use by participants in the intervention group (horizontal axis) and the total amount of their utterances (vertical axis). Boxes represent IQRs. White circles indicate the mean values. Black lines in boxes indicate the median values. Observations outside the first (third) quartile (i.e., outside of 1.5 × IQR) are indicated by black circles. No participant selected “4: never” for C and D.

Figure 4 shows the relationship between the digital device use of participants in the intervention group and their total number of utterances. In Figure 4A, we notice that participants who never used a computer had fewer utterances. In fact, the average number of utterances for participants who chose “4: never” was 86.50, compared to 108.32 for participants who chose options other than “4: never.” This difference was significant (*P*<.001). In Figure 4B, we observe that participants who usually used email had more utterances. In fact, the average number of utterances for participants who chose “1: usually” was 106.58, compared to 92.8 for participants who chose options other than “1: usually.” This difference was significant (*P*=.005). No significant associations were found between the frequency of smartphone use and flip phone use and the number of utterances (Figure 4C and 4D). This result persisted in regression analyses, even after controlling for age, gender, and education years. Associate regression coefficients were 19.69 (SE 4.15; *P*<.001) for computer use, 12.06 (SE 4.44; *P*=.01) for email use, 3.74 (SE 5.39; *P*=.49) for smartphone use, and −2.89 (SE 6.75; *P*=.67) for flip phone use.

## Discussion

### Overview of This Study

This paper presented the intervention effect of asking questions to improve cognitive function in healthy older adults. The intervention involved an RCT conducted at participants’ homes. There were no significant intervention effects on scores of TICS-J, recall tests, and verbal fluency tasks. Notably, the feasibility of the intervention was confirmed, as all participants were able to ask at least 1 question in every session, with no participants dropping out.

### Principal Results

This study identified no significant intervention effect in category fluency task scores and letter fluency task scores. In both types of verbal fluency tasks, the number of words produced per unit of time is commonly used as a behavioral index. However, the mechanisms of word production are supposedly quite different. The category fluency test requires retrieval of content included in a given semantic category, which involves access to semantic memory. This mechanism helps one use existing links between related concepts, such as those between the categorical label and its contents and among associated category members [38]. In contrast, the letter fluency task demands retrieval from a phonemic category in which the association of semantically related words should be suppressed [38]. This strategy depends on an effortful exploration of lexical systems. To ask questions, one must understand the meanings of others’ utterances and identify what is unclear with reference to one’s knowledge. A series of these processes would inevitably involve access to semantic memory.Nonetheless, no intervention effect was found on category verbal fluency and letter fluency.

This result may have been influenced by the short duration of the intervention. Other studies have involved longer experimental periods, lasting 6 [8] and 12 weeks [17]. Therefore, further studies are needed to clarify the effectiveness of asking questions, for example, by setting up a program with a longer intervention period. Another reason why the results did not show any significant intervention effects may pertain to various elements included in the conversations. We solely focused on asking questions and did not incorporate other elements into the intervention. Older adults who are more inclined to compare opinions with others display higher cognitive function [5]. Therefore, thinking about things from the perspective of others during conversations and thinking about the similarities and differences between oneself and others may be important for improving or maintaining cognitive function. Considering this, simply asking questions is not an adequately strong intervention to affect cognitive function, and combining questions with other cognitive elements, such as thinking about questions from the other person’s point of view or asking questions based on the similarities and differences between one’s thoughts and those of others, may be effective in strengthening future interventions.

Another area of interest is the feasibility of home-based interventions and their improvement. As shown in Table 4, all participants asked at least 1 question in every session, and no participants dropped out. These results indicate the feasibility of this intervention method.

The result that the frequency of device use was positively correlated with the number of questions asked during the intervention, even after controlling for age, gender, and years of education, suggests that future improvements to the usability of our system may enhance intervention effectiveness. One could be optimistic about this point. As Table 4 further indicates, the average number of utterances increased slightly more in the latter half of the intervention period compared to the former. This may have occurred because participants became accustomed to the system through repeated session participation. A more extended intervention period may compensate for the disadvantage owing to the lower frequencies of device use.

However, in terms of the social implementation of intervention programs, such a view may be too optimistic. This is because users may want to discontinue use of the program before becoming accustomed to it. From this perspective, we need to continue improving usability and developing appropriate evaluation methods, keeping in mind that our target population is older adults [39]. For example, it would be helpful to investigate which individuals, among those who use less frequently digital devices at baseline, are more likely to experience higher intervention effectiveness.

Furthermore, it would be important to have a perspective on what kind of interface to provide and how to personalize it according to the user’s preferences or personality as well as the characteristics of the user’s daily conversations [40], to keep them motivated to continue with the intervention program. This could lead to an overall increase in the effectiveness of the intervention, benefiting even those who are already accustomed to using the device.

### Strengths

This study has some strengths. One strength is that we presented a technical framework for examining the impact of “asking questions,” an important factor for conversation-based interventions for cognitive function, which was lacking in our previous studies [17,21]

Another strength of this study is that we successfully conducted home-based interventions while most intervention studies, including our previous ones [17,21], have been conducted on-site. Notably, this study demonstrated that home-based interventions are feasible even though some participants were unfamiliar with digital devices.

Finally, a strength of our study is that the system is fully automated after human-assisted training, requiring fewer human resources compared to previous methods. The impact of automation on participants’ satisfaction needs further investigation.

### Limitations

This study has several limitations. First, this study is limited by the diversity and sample size of the participants. Currently, this program only supports Japanese people; therefore, this study was conducted in an urban city in Japan for feasibility. Second, this study was conducted during the COVID-19 pandemic; hence, the research was designed to reduce communication as much as possible to keep the participants safe. To achieve this, we did not collect magnetic resonance imaging data from participants, unlike our previous intervention study [17]. Therefore, from a neural perspective, this study could not obtain useful information about the impact of asking questions on improving the intervention effect on cognitive function. The importance of asking questions in conversation-based intervention programs should be reevaluated in future research after accounting for these issues.

Finally, we did not sufficiently examine the effects of session topics and their order on the number of participants’ utterances. The topics suitable for improving cognitive function through conversation must be neither so challenging that participants cannot generate questions nor so easy that they do not train cognitive function. There has already been a study that identified the characteristics of utterances by older adults with higher cognitive functioning in group conversations, and then, identified conversation topics in which such utterances are likely to be observed [41]. However, this has not been examined in terms of encouraging participants to ask questions and enriching the conversation. Future studies should consider setting up efficient session themes for improving cognitive function.

### Conclusions

This study evaluated the possible improvements associated with introducing a dialogue-based robot in cognitive interventions, aiming to verify the training effects of asking questions in healthy older adults.

We did not observe any significant differences in global cognition between the 2 groups. The feasibility of our study was identified by (1) no loss in the intervention and (2) all participants asking at least 1 question in every session. We also recommend improvements to the intervention program, such as setting up more efficient session themes for cognitive training. This study has provided future directions for cognitive training studies of older adults at home.

## Supplementary material

10.2196/47229 Checklist 1 CONSORT (Consolidated Standards of Reporting Trials)-eHEALTH checklist (version 1.6.1).

## Abbreviations

CONSORT: Consolidated Standards of Reporting Trials
PICMOR: Photo-Integrated Conversation Moderated by Robots
RCT: randomized controlled trial
TICS-J: Telephone Interview for Cognitive Status-Japanese
WMS-R: Wechsler Memory Scale-Revised
